# One repeated transplantation of allogeneic umbilical cord mesenchymal stromal cells in type 1 diabetes: an open parallel controlled clinical study

**DOI:** 10.1186/s13287-021-02417-3

**Published:** 2021-06-10

**Authors:** Jing Lu, Shan-mei Shen, Qing Ling, Bin Wang, Li-rong Li, Wei Zhang, Duo-duo Qu, Yan Bi, Da-long Zhu

**Affiliations:** 1 Department of Endocrinology, Drum Tower Hospital Affiliated to Nanjing University Medical School, No 321, Zhongshan Road, Nanjing, 210008 Jiangsu China; 2 Clinical Stem Cell Center, Drum Tower Hospital Affiliated to Nanjing University Medical School, No 321, Zhongshan Road, Nanjing, 210008 Jiangsu China; 3 School of Clinical Medicine and Nursing, Suzhou Vocational Health College, No 28, Kehua Road, Suzhou International Education Park, Suzhou, 215151 Jiangsu China

**Keywords:** Mesenchymal stromal cells, Type 1 diabetes, Transplantation, β cell function

## Abstract

**Background:**

The preservation or restoration of β cell function in type 1 diabetes (T1D) remains as an attractive and challengeable therapeutic target. Mesenchymal stromal cells (MSCs) are multipotent cells with high capacity of immunoregulation, which emerged as a promising cell-based therapy for many immune disorders. The objective of this study was to examine the efficacy and safety of one repeated transplantation of allogeneic MSCs in individuals with T1D.

**Methods:**

This was a nonrandomized, open-label, parallel-armed prospective study. MSCs were isolated from umbilical cord (UC) of healthy donors. Fifty-three participants including 33 adult-onset (≥ 18 years) and 20 juvenile-onset T1D were enrolled. Twenty-seven subjects (MSC-treated group) received an initial systemic infusion of allogeneic UC-MSCs, followed by a repeat course at 3 months, whereas the control group (*n* = 26) only received standard care based on intensive insulin therapy. Data at 1-year follow-up was reported in this study. The primary endpoint was clinical remission defined as a 10% increase from baseline in the level of fasting and/or postprandial C-peptide. The secondary endpoints included side effects, serum levels of HbA1c, changes in fasting and postprandial C-peptide, and daily insulin doses.

**Results:**

After 1-year follow-up, 40.7% subjects in MSC-treated group achieved the primary endpoint, significantly higher than that in the control arm. Three subjects in MSC-treated group, in contrast to none in control group, achieved insulin independence and maintained insulin free for 3 to 12 months. Among the adult-onset T1D, the percent change of postprandial C-peptide was significantly increased in MSC-treated group than in the control group. However, changes in fasting or postprandial C-peptide were not significantly different between groups among the juvenile-onset T1D. Multivariable logistic regression assay indicated that lower fasting C-peptide and higher dose of UC-MSC correlated with achievement of clinical remission after transplantation. No severe side effects were observed.

**Conclusion:**

One repeated intravenous dose of allogeneic UC-MSCs is safe in people with recent-onset T1D and may result in better islet β cell preservation during the first year after diagnosis compared to standard treatment alone.

**Trial registration:**

ChiCTR2100045434. Registered on April 15, 2021—retrospectively registered, http://www.chictr.org.cn/

**Supplementary Information:**

The online version contains supplementary material available at 10.1186/s13287-021-02417-3.

## Introduction

Type 1 diabetes (T1D) is an autoimmune disease characterized by a progressive and irreversible loss of insulin-producing β cells. Once diagnosed, individuals with T1D require life-long injections of exogenous insulin. Although intensive insulin therapy allows for improved glucose homeostasis, it cannot replace the highly specialized work done by pancreatic islets, neither can it suppress the autoimmune destruction of residual β cells. Preserved residue β cell function is associated with attenuation of glycemic fluctuation, as well as decreased risks of chronic diabetic complications [1, 2]. Therefore, blocking autoimmune attack, preserving, or even restoring the endogenous β cell function has been a long sought-after solution for T1D.

Mesenchymal stromal cells (MSCs) are non-hematopoietic, multipotent cells that can be sourced from various tissues, including bone marrow, umbilical cord (UC), and adipose tissue. Due to their properties of plasticity, immunomodulatory, anti-inflammatory, and homing ability to injured sites, MSCs are well studied as a cell-based therapy for tissue degeneration and immune-based pathologies, such as graft-versus-host disease, osteogenesis, cardiac disease, and Crohn’s disease [3–5]. Additionally, MSCs are easily expandable for generating sufficient cell numbers, immune privileged from alloreactivity and less ethically controversial compared to other types of stem cells; therefore, they are widely used in over 6000 clinical trials [6, 7].

Plenty of evidence including our previous study in animal models of T1D have demonstrated that transplantation with MSCs can delay T1D onset, reverse hyperglycemia after onset, and increase both β cell function and mass [8–12]. Our work further clarified that MSCs predominantly accumulated in pancreatic tissues at 28 days after systemic infusion in none obese diabetic (NOD) mice. MSC infusion significantly attenuated insulitis at least in part through modulating the balance between pro- and anti-inflammation, as well as effector T and regulatory T cells. Recently, clinical trials are undertaken to investigate the effect of MSCs in people with T1D. Up to now, two randomized controlled trials (RCT) have been published, both of which used single intravenous transplantation [13, 14]. No side effect was reported, and β cell function was preserved or even increased as compared with regular insulin therapy. However, insulin independence was not achieved in most individuals received MSCs in these studies.

According to our previous data, the effects of MSCs on preserving β cell function and modulating inflammation were dose-dependent in diabetic NOD mice, and multiple doses of MSCs prolonged their presence in the pancreatic tissues and displayed longer effects [9]. Moreover, in our pilot trial of MSC transplantation for T1D, the levels of C-peptide in recipients who received a single dosage of MSCs showed a decreased tendency after three months (data not shown). Therefore, we decided to modify the MSC-based immunotherapy and increased the treatment course to repeated transplantation with an interval of three months. The objective of the present study was to evaluate the safety and efficacy of one repeated allogenous UC-MSCs in treatment of subjects with T1D.

## Methods

### Study design and participants

This is an open-label, parallel-arm, non-randomized study comparing MSCs intervention with standard insulin therapy in participants with T1D at the Endocrinology Department of Nanjing Drum Tower Hospital during 2013–2019. Individuals with T1D were diagnosed based on clinical features with at least one positive islet antibody and/or a fasting C-peptide ≤ 200 pmol/L. All participants and/or guardians provided written informed consent. The study was approved by both the Ethical and Scientific Committee of our hospital and the Office of Science and Technology in the hospital supervised our data management, trial monitoring and safety. This study was registered as a clinical trial (ChiCTR2100045434, www.chictr.org.cn).

Eligible participants were aged 8 to 55 years at enrollment with insulin requirement since diagnosis of T1D and had a level of fasting C-peptide ≥ 100 pmol/L. Exclusions included cardiorespiratory insufficiency, failure of one or more organs, positive for HIV or viral hepatitis, pregnancy, and an underlying hematologic, rheumatic, psychiatric, or malignant disease. Patients who attended our clinic during 2013–2019 were assessed and enrolled if eligible. Participants consenting to receive UC-MSC therapy were assigned to the intervention group while the others were assigned to the control group.

### Preparation of UC-MSC

The MSCs were isolated from Wharton’s Jelly of UC using the tissue explant method in GMP-certified facilities [6]. Briefly, fresh UCs were obtained from informed healthy mothers in maternity department after normal deliveries. The UCs were cut into pieces of 1 mm^3^ and incubated in a 5% CO_2_ incubator at 37 °C and culture medium was changed every 3–4 days. When well-developed colonies of fibroblast-like cells appeared, the cells were trypsinized and transferred into a new flask for further expansion. Both UCs and isolated fibroblast-like cells were cultured in low glucose Dulbecco’s modified Eagle’s medium containing 10% fetal bovine serum, 1% penicillin/streptomycin, and 1.25 mg/ml amphotericin B.

MSCs were identified as described before [6], and in accordance with three definition criteria: (i) being spindle-like and adhesion to plastic, (ii) positive expression of (> 95%) CD73, CD90, and CD105, negative expression (< 2%) for CD19, CD34, CD11b, CD45, and HLA-DR, and (iii) multilineage differentiation potentials of adipogenesis, osteogenesis, and chondrogenesis. All cells were harvested in passages 2–4 and cryopreserved. Before being cryopreserved, the samples of supernatant and cells were sent to the quality control lab for inprocess testing. Before clinic use, UC-MSCs were immediately unfrozen in thermocell mixing block, and then washed with complete medium by centrifugation at 1200 n/min for 5 min followed by resuspension in fresh complete medium. After 24 h of culture, the complete medium was changed again to ensure elimination of the cryoprotective agent. Then, the cells were passaged two times and underwent a fast release testing as last safeguard measure to ensure the safety of recipients. The release assays included sterility, endotoxin, mycoplasma, cell amount and viability, and BSA residual level. MSCs were finally released for infusion with a viability of > 90% and absence of contamination. Besides, chromosomal karyotype of UC-MSC was defined to ensure sex-matched donors for recipients.

### Interventions

All participants received intensive insulin therapy, either with multiple-dose insulin injection regimen or continuous subcutaneous insulin infusion. T1D education was offered to all subjects, including the principles of flexible insulin therapy. Participants were instructed to perform self-monitored blood glucose and adjust insulin dose accordingly. Target levels of fasting blood glucose were 5.0–8.0 mmol/L for juveniles and 4.0–7.2 mmol/L for adults, while levels of postprandial blood glucose were suggested to be no more than 10.0 mmol/L for all.

Participants in the MSC-treated group received repeat transplantation with an interval of 3 months. All the female participants received the same batch of UC-MSC from a female donor while the male participants received the batch from a male donor. At each time, 1.0 × 10^6^ cells/kg was given as one single intravenous infusion within 10 min. To prevent potential risk of hypersensitivity, 2.5 mg dexamethasone was injected intravenously prior to MSC infusion. Since the first transplantation, participants were followed up at 3, 6, and 12 months and yearly afterward. Adverse events and their severity were assessed and recorded throughout the study.

### Endpoints and assessments

The primary efficacy endpoint was clinical remission characterized by improved β cell function through 1-year follow-up. Accordingly, clinical remission was defined as a 10% increase from baseline in the levels of fasting and/or postprandial C-peptide. Secondary efficacy endpoints included changes in HbA1c, fasting C-peptide, 2-h postprandial C-peptide, and daily insulin dose, as well as safety of the therapy.

During each visit, participants underwent history taking and physical examinations; meanwhile, daily insulin doses were recorded. Besides, blood samples were obtained for laboratory tests. Standard-meal tolerance test was performed for islet function evaluation. Briefly, after a 10-h overnight fast, each participant received steamed bread which was made of flour with 100 g for adult and 2.3 g/kg up to 100 g for child. The levels of plasma glucose and C-peptide before and 2 h after food stimulation were analyzed. HbA1c was measured by high-pressure liquid chromatography. Islet autoantibodies including insulin autoantibody, glutamic acid decarboxylase autoantibody, and insulinoma-associated antigen-2 autoantibody were determined by ELISA technique, while islet cell antibody was determined by indirect immunofluorescence. Whole blood cell counts, liver and renal function, infections, and cancer screening were performed for safety assessment.

### Statistical analysis

All statistical analyses were performed using SPSS version 22.0 (SPSS Inc., USA). Descriptive statistics were reported as mean ± SD for normally distributed variables or median (interquartile range [IQR]) for data of skewed distribution. Continuous variables were compared by the Student’s t test or Mann-Whitney U test, and categorical variables were compared by the chi-square analysis or Fisher exact test. The main analysis was done on the per-protocol participant dataset, which comprised subjects who received the allocated treatment and completed follow-up. Correlation analyses were performed using the Pearson or Spearman tests according to variable characteristics and distribution. Multivariable logistic regression analyses were used to assess the associations of clinical characteristics at baseline with final achievement of clinical remission. The odds ratios (ORs) and 95% confidence intervals (CIs) were given.

## Results

### Characteristics of the participants

**Fig. 1:**
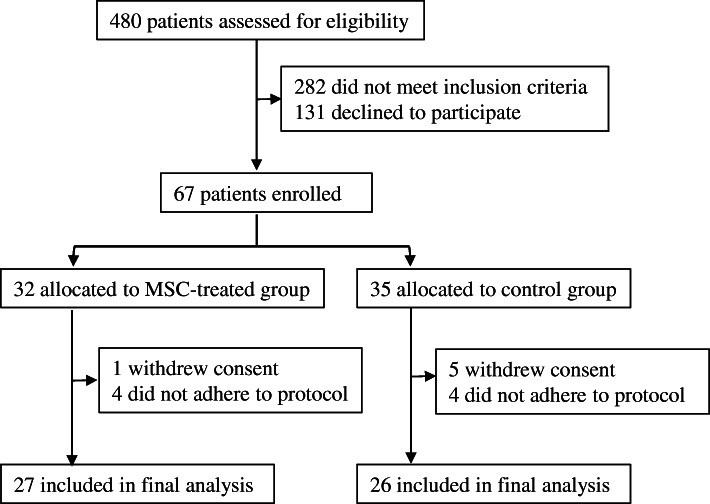
Trial profile

Sixty-seven participants were enrolled with 32 assigned to the MSC-treated group and 35 to the control group. During the trial 5 participants in the MSC-treated group and 9 participants in the control group discontinued treatment because of none-adherence to study protocol or withdrawal of consent (Fig. 1). During the trial, five participants in the MSC-treated group discontinued treatment: four individuals did not adhere to study protocol due to personal reasons and failed to complete follow-up schedule (two individuals discontinued after 3 months before the second dose and two patients after 5 months), and one withdrew her consent on the day before first transplantation. Nine participants in the control group discontinued the trial: five withdrew their consent within 3 months and four individuals adhered to follow-up schedule no more than half a year. A total of 53 participants (53% male, 62% adult) were included for final analysis. The mean age was 24.9 ± 14.2 years, and the mean duration of time from diagnosis of T1D to enrolment of this study ranged from 0 to 24 month (4.7 ± 6.1 months). All participants presented obvious symptoms of hyperglycemia at disease onset, and 62.3% of them complicated with diabetic ketosis or diabetic ketoacidosis (DKA). The mean body mass index (BMI) was 18.9 ± 3.5 kg/m^2^,^,^ and the mean HbA1c level was 9.5 ± 2.9%. Insulin therapies were immediately started once diabetes was diagnosed. Demographic and baseline characteristics were similar between the MSC-treated and control groups (Table 1), with the exception of daily insulin dose which was lower in the MSC-treated group.

**Table 1:** Baseline characteristics of the participants

	Control (*n* = 26)	MSC-treated (*n* = 27)	*P*
Sex (male/female)	13/13	12/15	0.685
Age (years)	27.4±14.3	22.4±13.9	0.207
Duration (months)	1.0 (0.1, 7.6)	2.3 (0.9, 7.7)	0.068
DK/DKA (n)	16	17	0.915
BMI (kg/m^2^)	19.1±3.5	18.7±3.6	0.700
HbA1c (%)	10.0 ± 3.0	9.0±2.8	0.174
Islet antibodies (n)			0.576
Negative	2	3	
One antibody	8	10	
≥ 2 antibodies	16	14	
FCP (pmol/L)	242.5±91.7	238.0±117.0	0.877
PCP (pmol/L)	574.0±328.0	567.4±419.0	0.950
Insulin doses (IU/kg/day)	0.48±0.24	0.35±0.14	0.020

Data are shown as number (n), means ± S.D or median (interquartile range). DK, diabetic ketosis; FCP, fasting C-peptide; PCP, 2-h postprandial C-peptide

### Adverse events

Three adult recipients had mild fever which occurred 1.5, 17, and 20 h after MSC infusion respectively, and the highest temperature was no more than 38.3 °C. Two of them occurred at the second time of transplantation, while another experienced at the first transplantation. The fever was self-limiting, and all of them recovered within 24-h of MSC treatment without any antipyretics or antibiotics. Other recipients tolerated the procedure well. There was no severe adverse effect, such as mortality, thrombosis, severe infection, or allergic reactions. During the first year, no tumors or chronic infections have been diagnosed in any of the participants. Neither did any of them reported episodes of hyperglycemic ketoacidosis or assisted hypoglycemia. Besides, the four withdrawn recipients who received one or two doses of MSCs as declared above reported no adverse events during their available follow-up.

### Efficacy evaluation

At the end of 1-year follow-up, 11 recipients in MSC-treated group maintained clinical remission (11/27, 40.7%) whereas the control group had a significantly lower rate of clinical remission (3/26, 11.5%, *p* = 0.041). A post hoc power analysis indicated 68.4% power to assess such a difference in clinical remission. Besides, none of the control group but three MSC-treated adults achieved insulin free, although there was no statistic difference. Two of the three MSC-treated adults became insulin independent within 3 months after the 1st transplantation and initiated insulin again 1 year and 20 months later respectively. Another recipient achieved insulin free 6 months after the 2nd transplantation and started again 3.8 months later.

**Fig. 2:**
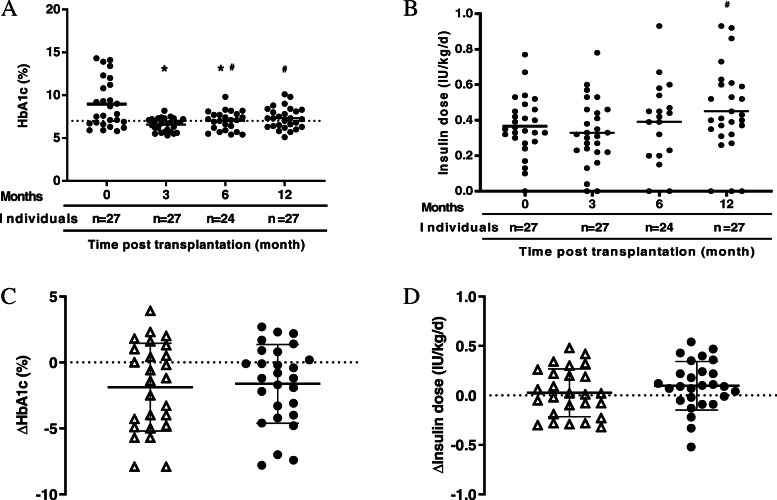
Changes of HbA1c and exogenous insulin requirement during 1-year follow-up. Levels of HbA1c (**A**) and doses of daily insulin (**B**) in MSC-treated recipients. Data are expressed as individual values. *p < 0.05 compared with pre-transplantation, # *p* < 0.05 compared with 3-month post-transplantation. Absolute changes in HbA1c (**C**) and insulin dose (**D**) between baseline and 1-year after follow-up for control (open triangles) and MSC-treated subjects (closed circles). Data are expressed as means ± SEM

**Fig. 3:**
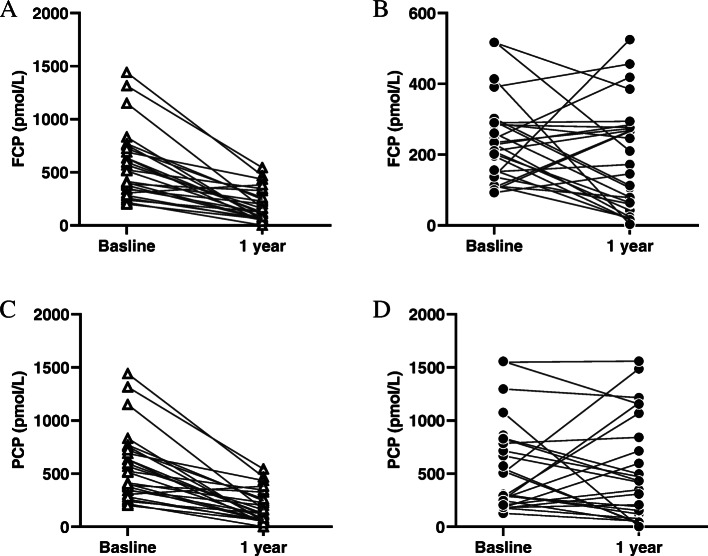
C-peptide response to a standard-meal tolerance test at baseline and 1-year follow-up. Fasting C-peptide concentrations for individual subjects in control (**A**) and MSC-treated group (**B**). Two-hour response of C-peptide concentrations for individual subjects in control (**C**) and MSC-treated group (**D**). FCP, fasting C-peptide; PCP, postprandial C-peptide

HbA1c levels were significantly decreased and reached their lowest levels at 3 months (6.6 ± 0.8%) in the MSC-treated group, but gradually increased to 6.9 ± 1.1% and 7.3 ± 1.3% respectively at 6 and 12 months. The average daily insulin dose requirements in MSC-treated individuals were relatively stable during the follow-up. Generally, there were no differences in the changes of HbA1c or insulin doses between the control and MSC-treated groups (Fig. 2). Residue β cell function was evaluated by standard-meal tolerance test. Although MSC-treated group showed attenuated reduction in fasting and postprandial C-peptide, no statistic difference was observed (Additional file 1: Table S1). For individual subjects in MSC-treated group, 11 out of 27 subjects increased in fasting C-peptide and 10 of them increased in postprandial C-peptide. On the other side, increased fasting and postprandial C-peptide were seen in only 6 and 5 of the 26 control subjects respectively (Fig. 3).

Considering the heterogeneity in T1D, we re-evaluated residue β cell function in subgroups based on the onset of age (Additional file 1: Table S1). Demographic characteristics and levels of HbA1c, FCP and PCP were comparable between the MSC-treated and control groups in both adults and juveniles (data not shown). Among adult-onset T1D (≥ 18 years), the mean level of postprandial C-peptide decreased in the control subjects but increased in MSC-treated subjects. After adjusting for baseline levels, the ratio of changed postprandial C-peptide was significantly increased in MSC-treated group compared with the ratio of change in the control group. On the other hand, MSC treatment did not show protective effect on β cell function in juveniles. Subgroup analysis based on the onset with or without diabetic ketoacidosis or DKA was also assessed and showed similar tendency to the analysis of the whole study population (Additional file 2: Table S2).

### Factors associated with the efficacy of clinical remission

Clinical remission occurred in both the control and MSC-treated groups; thus, contributory factors were first analyzed in the whole study population. Intriguingly, multivariable logistic regression analysis further confirmed that subjects treated with MSC were four times more likely to achieve clinical remission than those with regular insulin therapy (Additional file 3: Table S3; OR = 4.38, *p* = 0.044). Besides, subjects with higher BMI (OR = 1.26, *p* = 0.038) and lower baseline fasting C-peptide (OR = 0.99, *p* = 0.046) were more likely to become clinical remission.

**Fig. 4:**
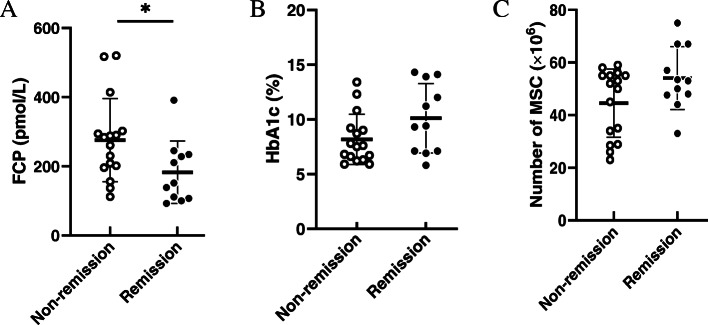
Comparisons of baseline C-peptide, HbA1c, and transplanted MSCs among recipients achieving clinical remission or not. Fasting C-peptide (**A**) and HbA1c (**B**) at baseline, and numbers of transplanted MSC (**C**) between recipients who achieved (closed circles) and did not achieve (open circles) clinical remission. Data are expressed as means ± SEM. *p < 0.05 compared with non-remission subjects

To further examine potential factors contributing to the efficacy of MSC treatment, all individuals receiving MSC were grouped according to the achievement of clinical remission. Compared with recipients who did not achieve remission at 1-year follow-up, those achieved remission had significantly lower levels of fasting C-peptide at baseline (*p* = 0.040), and relatively higher levels of HbA1c and infused numbers of MSC though without statistical significance (Fig. 4). A logistic regression analysis was applied of fasting C-peptide, HbA1c, and cell numbers of MSC; meanwhile, potential confounders (age, duration, sex, BMI) were included and adjusted. According to the step-wise forward selection, fasting C-peptide (OR 0.982(0.966–0.997), *P* = 0.023) and number of transfused MSCs (OR 1.141(1.017–1.280), *P* = 0.025) were independently associated with the achievement of clinical remission.

## Discussion

This is the first study to report the feasibility and general tolerance of one repeated systemic UC-MSC transfusion in both juveniles and adults with T1D. After 1-year follow-up, 40.7% participants remained clinical remission in MSC-treated group, nearly 2.5 times higher than those achieved in the control group, indicating the efficacy of UC-MSCs in preservation of β cell function.

Natural history studies in T1D have shown a progressive decline in insulin secretion ever since the pre-clinical stage and continuing after diagnosis [15]. During the 1st year of diagnosis, only about 20% of individuals with T1D maintained C-peptide, and mean levels of C-peptide slide down across all age groups, with 17.6% decrease in adults of 21–46 years and almost 40% decrease in youth of 7–12 years old [16]. Retention of endogenous β cell function is associated with beneficial outcomes but still challengeable in clinic. Autologous nonmyeloablative hematopoietic stem cell transplantation (AHSCT) is the first approach ever proved to successfully halt disease process. In a multicenter analysis, 59% of individuals with T1D achieved insulin free within 6 months of transplantation. The median duration of insulin independence was 12 months [17], and the longest lasting time was 5 years in our center [18]. Nevertheless, 52% of treated individuals experienced adverse effects with one serious case who died of Pseudomonas aeruginosa sepsis [14]. Therefore, AHSCT is not widely applied and safer cell-based therapeutic options are in demand.

MSCs also have unique immunomodulatory properties and represent the most clinically studied experimental cell therapy in many immune-mediated disorders, such as graft-versus-host disease, Crohn’s disease, and systemic lupus erythematosus [2, 19]. Based on encouraging pre-clinical animal data in T1D models, MSCs also have become an attractive potential therapy for T1D. Up to now, 15 trials have been registered on the website of ClinicalTrial.gov. Regimens of MSC treatment including cell origin, dose, frequency and route of transplantation are varied among registered clinical centers.

In our previous pilot trial, four individuals with T1D received a single transfusion of allogenic bone marrow MSCs [9]. No side effect was observed during 4-year follow-up. One responder showed increased postprandial C-peptide during the first year and the other achieved insulin independence for 3 months. MSC treatment appeared to be safe and beneficial for β cell preservation, which was contemporaneously confirmed by an open-label RCT in Sweden [13]. Twenty newly diagnosed individuals with T1D were randomly assigned to either autologous bone marrow MSCs or insulin-only treatment. After 1 year, a mean 5% increase in the peak C-peptide response and 10% increase in the area under the curve of C-peptide was observed in MSC-treated subjects, significantly higher than the percent changes of C-peptide in the control group. But no case of insulin free was reported. In an earlier double blind RCT, 15 of 29 newly diagnosed T1D received a single transfusion of allogenic UC-MSCs and displayed an obviously increased and constantly higher fasting C-peptide than the control group through 2-year follow-up. Three recipients discontinued insulin 1 year after transplantation, but little information was available for the lasting period [14].

In the above-mentioned reports, preserved C-peptide appeared to be a consistent efficacy, whereas insulin independence seemed less achievable; therefore, we defined clinical remission in this study to be over 10% increase in either fasting or postprandial C-peptide. Based on an expended sample size and a parallel controlled design, our study demonstrated a significantly higher achievement of clinical remission after one repeated UC-MSC transfusion. Moreover, insulin independence otherwise not the primary endpoint was observed in three recipients in contrast to none of the control group, thus further implying the effect of our MSC therapeutic strategy in improving endogenous insulin secretion. The difference in the achievement of insulin independence between our and previous trials is probably due to disparities in study design, characteristics of enrolled subjects, source, and dose of MSCs. Future RCTs with expanded sample size may take insulin independence as a primary endpoint to further address the efficacy of MSC therapy in applicable subsets of T1D.

Among secondary endpoints, changes of C-peptide showed distinct tendencies between the groups. Levels of C-peptide decreased in most participants in the control group, with a mean 28% and 32% decrease in the fasting and postprandial state, respectively. On the other hand, a convergence for improved C-peptide in MSC-treated group was observed, with a mean 9% decrease in fasting C-peptide and 16% increase in postprandial C-peptide, although no statistical significance was achieved. Glycemic control maintained around the targeted level without obviously increased insulin dose in participants received MSCs. Nevertheless, such a benefit in glycemic control was not restricted to MSC intervention, because no difference was observed in levels of HbA1c or dose of insulin when compared to the control group. This founding was consistence with that in the RCT from Sweden [13], probably due to comparable basic diabetic care provided to all participants.

Intravenous administration of MSCs is generally considered to be clinical safe, without side effects recorded in previous trials in T1D [20]. In this study, we increased the treatment course to repeated transfusion of MSCs. Three recipients experienced mild fever but recovered spontaneously within 24 h. Such a post-infusion febrile reaction was once reported in clinical trials of other diseases [21]. Notably, we did not observe any severe adverse events, and both juvenile and adult recipients tolerated well.

There was a heterogenicity in the therapeutic efficacy of UC-MSCs, which addressed the potential factors associated with therapeutic effects of UC-MSCs for T1D. According to previous experiences in AHSCT, good responders were older at onset, were treated soon after T1D diagnosis, had lower instances of DKA, and had lower HbA1c levels at the time of transplantation [22]. However, there was no difference in age of onset, gender, occurrence of DKA, or duration of T1D between good and poor responders to UC-MSC treatment in this trial. In contrast, we found that subjects who had lower levels of fasting C-peptide at the time of enrollment and relatively higher HbA1c levels were more likely to achieve clinical remission, indicating that UC-MSCs are beneficial for newly diagnosed T1D with less residual β cell function and relatively poor glycemic control. As a technical element, the number of transfused UC-MSCs also influenced the therapeutic outcomes. In our model for predicting clinical remission, the number of UC-MSCs exerted larger contribution than other parameters, reinforcing the evidence of dose-dependent efficacy of MSC treatment. The optimal doses as well as courses of MSC transplantation should be further investigated in future studies.

The present study has some limitations. Firstly, there was an imbalance in insulin requirement at baseline between the two groups, indicating the possibility of selection bias due to non-randomized design. Since this trial took relatively long time to complete, it may further introduce discordance or confounding into data collection. We adjusted potential confounders while analyzing contributors to clinical remission of T1D, but confounding related to unknown variables might still exist. Secondly, in the standard-meal tolerance test for the evaluation of β cell function, we only provided levels of C-peptide before and 2 h after meal. Because multiple blood samplings during the tolerance test was unacceptable by some participants especially young children, levels of C peptide at additional time points or area under the curve were not available. Thirdly, a post hoc power analysis indicated 68.4% power to assess the effect of MSCs on clinical remission. Future RCTs with larger sample size and an intention-to-treat analysis are required to prove the efficacy of MSCs with adequate power.

## Conclusion

This trial is the first demonstration, to our knowledge, one repeated intravenous dose of allogeneic UC-MSCs is feasible and safe for individuals with T1D. Besides, it provides evidence that this therapy may be beneficial for preservation of endogenous β cell function during the first year after diagnosis compared to standard treatment alone. Larger RCTs with longer follow-up are required for further validation, and the treatment regimen requires stepwise optimization for better efficacy.

## Supplementary Information

**Additional file 1: Table S1.** Absolute and percent changes in β cell function at 1-year for the total population and age-based subgroups. Data are shown as means ± S.D. FCP, fasting C-peptide; PCP, 2-h postprandial C-peptide. ΔFCP ratio was defined as the calibration of changed FCP at 1-year follow-up by its baseline level; ΔPCP ratio was defined as the calibration of changed PCP at 1-year follow-up by its baseline level.

**Additional file 2: Table S2.** Absolute and percent changes in β cell function at 1-year for subgroups based on DK/DKA history. Data are shown as means ± S.D. DK, diabetic ketosis; DKA, diabetic ketoacidosis.

**Additional file 3: Table S3.** Logistic regression analysis of clinical characteristics associated with clinical remission at 1-year for the total population.

## Data Availability

The datasets generated during and/or analyzed during the current study are available from the corresponding author on reasonable request.

## Abbreviations

T1D: Type 1 diabetes
MSCs: Mesenchymal stromal cells
UC: Umbilical cord
NOD: None obese diabetic
RCT: Randomized controlled trials
DKA: Diabetic ketoacidosis
BMI: Body mass index
IQR: Interquartile range
ORs: Odds ratios
CIs: Confidence intervals
AHSCT: Hematopoietic stem cell transplantation
