# Correction: One repeated transplantation of allogeneic umbilical cord mesenchymal stromal cells in type 1 diabetes: an open parallel controlled clinical study

**DOI:** 10.1186/s13287-025-04563-4

**Published:** 2025-08-06

**Authors:** Jing Lu, Shan-mei Shen, Qing Ling, Bin Wang, Li-rong Li, Wei Zhang, Duo-duo Qu, Yan Bi, Da-long Zhu

**Affiliations:** 1 Department of Endocrinology, Drum Tower Hospital Affiliated to Nanjing University Medical School, No 321, Zhongshan Road, Nanjing, 210008 Jiangsu China; 2 Clinical Stem Cell Center, Drum Tower Hospital Affiliated to Nanjing University Medical School, No 321, Zhongshan Road, Nanjing, 210008 Jiangsu China; 3 School of Clinical Medicine and Nursing, Suzhou Vocational Health College, No 28, Kehua Road, Suzhou International Education Park, Suzhou, 215151 Jiangsu China

**Correction: Stem Cell Research & Therapy**

10.1186/s13287-021-02417-3

The original article presents an error in Fig. 3 whereby the chart in Fig. 3A is a duplicate of the chart in Fig. 3C.

The image for Fig. 3A has been corrected and can be viewed ahead in this correction article.

**Fig. 3:**
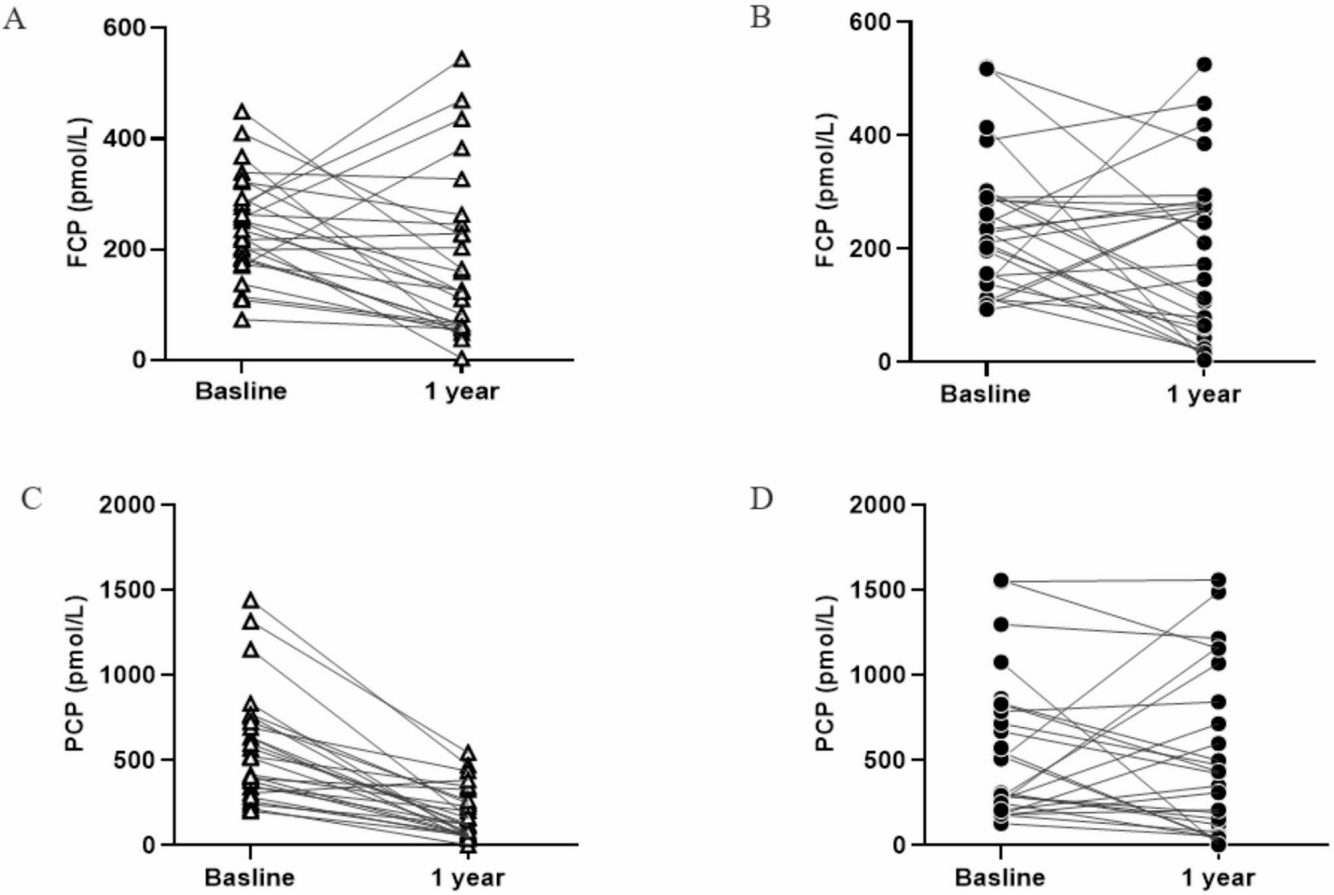
C-peptide response to a standard-meal tolerance test at baseline and 1-year follow-up for control (open triangles) and MSC-treated subjects (closed circles). Fasting C-peptide (FCP) concentrations for all individuals in control (**A**) and MSC-treated group (**B**). Two-hour response of C-peptide concentrations for the same individuals in control (**C**) and MSC-treated group (**D**)

The authors are confident that this error does not compromise the study design, analysis, interpretation or conclusions.

