# Supplementary material for: One repeated transplantation of allogeneic umbilical cord mesenchymal stromal cells in type 1 diabetes: an open parallel controlled clinical study
Source: Stem Cell Res Ther. 2021 Jun 10;12:340. doi: 10.1186/s13287-021-02417-3 (PMC8194026; doi:10.1186/s13287-021-02417-3)
Supplement: Supplementary file 1 — Additional file 1: Table S1. Absolute and percent changes in β cell function at 1-year for the total population and age-based subgroups. Data are shown as means ± S.D. FCP, fasting C-peptide; PCP, 2-h postprandial C-peptide. ΔFCP ratio was defined as the calibration of changed FCP at 1-year follow-up by its baseline level; ΔPCP ratio was defined as the calibration of changed PCP at 1-year follow-up by its baseline level. [file 13287_2021_2417_MOESM1_ESM.docx]

Additional file 1: Table S1.

Table S1. Absolute and percent changes in β-cell function at 1-year for the total population and age-based subgroups

|  | Total population | | |  | Adults | | |  | Juveniles | | |
| --- | --- | --- | --- | --- | --- | --- | --- | --- | --- | --- | --- |
|  | control | MSC-treated | *P* |  | control  (n=18) | MSC-treated  (n=15) | *P* |  | control  (n=8) | MSC-treated  (n=12) | *P* |
| ΔFCP | -78.4 ± 154.0 | -52.5 ± 166.8 | 0.561 |  | -86.2 ± 168.6 | -7.1 ± 178.9 | 0.201 |  | -60.8 ± 123.3 | -109.3 ± 136.7 | 0.430 |
| ΔPCP | -209.3 ± 282.6 | -35.9 ± 448.2 | 0.103 |  | -231.0 ± 232.0 | 22.9 ± 531.5 | 0.102 |  | -160.4 ± 387.9 | -124.1 ± 286.0 | 0.822 |
| ΔFCP ratio | -28.0 ± 58.2 | -9.0 ± 81.7 | 0.336 |  | -33.2 ± 57.8 | 16.4 ± 93.8 | 0.072 |  | -16.3 ± 61.4 | -40.7 ± 51.2 | 0.347 |
| ΔPCP ratio | -32.5 ± 56.6 | 16.2 ± 114.7 | 0.064 |  | -42.2 ± 34.4 | 39.8 ± 130.0 | 0.031* |  | -10.6 ± 88.2 | -19.1 ± 80.4 | 0.834 |

Data are shown as means ± S.D. ΔFCP ratio was defined as the calibration of changed FCP at 1-year follow-up by its baseline level; ΔPCP ratio was defined as the calibration of changed PCP at 1-year follow-up by its baseline level. FCP, fasting C-peptide; PCP, 2-hour postprandial C-peptide.
