# Supplementary material for: One repeated transplantation of allogeneic umbilical cord mesenchymal stromal cells in type 1 diabetes: an open parallel controlled clinical study
Source: Stem Cell Res Ther. 2021 Jun 10;12:340. doi: 10.1186/s13287-021-02417-3 (PMC8194026; doi:10.1186/s13287-021-02417-3)
Supplement: Supplementary file 2 — Additional file 2: Table S2. Absolute and percent changes in β cell function at 1-year for subgroups based on DK/DKA history. Data are shown as means ± S.D. DK, diabetic ketosis; DKA, diabetic ketoacidosis. [file 13287_2021_2417_MOESM2_ESM.docx]

Additional file 2: Table S2.

Table S2. Absolute and percent changes in β-cell function at 1-year for subgroups based on DK/DKA history

|  | Total population | | |  | without DK/DKA | | |  | DK/DKA | | |
| --- | --- | --- | --- | --- | --- | --- | --- | --- | --- | --- | --- |
|  | control | MSC-treated | *P* |  | control  (n=10) | MSC-treated  (n=10) | *P* |  | control  (n=16) | MSC-treated  (n=17) | *P* |
| ΔFCP | -78.4 ± 154.0 | -52.5 ± 166.8 | 0.561 |  | -74.6 ± 163.2 | -108.8 ± 175.1 | 0.657 |  | -80.7 ± 153.4 | -19.4 ± 157.6 | 0.267 |
| ΔPCP | -209.3 ± 282.6 | -35.9 ± 448.2 | 0.103 |  | -225.0 ± 268.8 | -149.3 ± 537.8 | 0.698 |  | -199.5 ± 299.1 | 27.9 ± 393.8 | 0.076 |
| ΔFCP ratio | -28.0 ± 58.2 | -9.0 ± 81.7 | 0.336 |  | -30.6 ± 57.3 | -29.1 ± 80.3 | 0.962 |  | -26.3 ± 60.6 | 2.9 ± 82.6 | 0.258 |
| ΔPCP ratio | -32.5 ± 56.6 | 16.2 ± 114.7 | 0.064 |  | -43.3 ± 38.1 | -18.4 ± 87.3 | 0.424 |  | -25.7 ± 65.9 | 35.8 ± 126.0 | 0.097 |

Data are shown as means ± S.D. DK, diabetic ketosis; DKA, diabetic ketoacidosis.
