# Supplementary material for: One repeated transplantation of allogeneic umbilical cord mesenchymal stromal cells in type 1 diabetes: an open parallel controlled clinical study
Source: Stem Cell Res Ther. 2021 Jun 10;12:340. doi: 10.1186/s13287-021-02417-3 (PMC8194026; doi:10.1186/s13287-021-02417-3)
Supplement: Supplementary file 3 — Additional file 3: Table S3. Logistic regression analysis of clinical characteristics associated with clinical remission at 1-year for the total population. [file 13287_2021_2417_MOESM3_ESM.docx]

Additional file 3: Table S3.

Table S3. Logistic regression analysis of clinical characteristics associated with clinical remission at 1-year for the total population

|  | Univariate analysis | | Multivariate analysis | |
| --- | --- | --- | --- | --- |
| variables | OR (95% CI) | *P* value | OR (95% CI) | *P* value |
| MSC group | 5.605 (0.918-34.226) | 0.062 | 4.380 (1.042-18.412) | 0.044 |
| Gender | 0.578 (0.102-3.275) | 0.536 | 1.053 (0.902-1.228) | 0.515 |
| Age | 0.992 (0.926-1.063) | 0.824 | 0.992 (0.926-1.063) | 0.824 |
| Duration | 1.061 (0.895-1.259) | 0.495 | 1.033 (0.899-1.188) | 0.645 |
| BMI | 1.377 (1.030-1.842) | 0.031 | 1.258 (1.013-1.563) | 0.038 |
| HbA1c | 1.449 (1.022-2.054) | 0.037 | 1.304 (0.986-1.726) | 0.063 |
| FCP | 0.992 (0.983-1.001) | 0.093 | 0.992 (0.983-1.000) | 0.046 |
| Insulin dose | 0.053 (0.000-5.628) | 0.217 | 0.047 (0.001-3.833) | 0.174 |
